# Sensitive detection of EBV microRNAs across cancer spectrum reveals association with decreased survival in adult acute myelocytic leukemia

**DOI:** 10.1038/s41598-019-56472-1

**Published:** 2019-12-30

**Authors:** Mercedeh Movassagh, Cliff Oduor, Catherine Forconi, Ann M. Moormann, Jeffrey A. Bailey

**Affiliations:** 1 Department of Bioinformatics and Integrative Biology, University of Massachusetts Medical School, Worcester, MA USA; 2 Center for Global Health Research, Kenya Medical Research Institute, Kisumu, Kenya; 3 Department of Biomedical Sciences and Technology, Maseno University, Maseno, Kenya; 4 Department of Medicine, University of Massachusetts Medical School, Worcester, MA USA; 5 Department of Pathology and Laboratory Medicine, Warren Alpert Medical School, Brown University, Providence, RI USA

**Keywords:** Cancer genomics, Data mining

## Abstract

Epstein Barr virus (EBV) is the etiologic agent involved in numerous human cancers. After infecting the host, EBV establishes a latent infection, with low levels of messenger RNA (mRNA) and protein expression, evolved to evade immune recognition. Conversely, EBV microRNAs (miRNA) are expressed ubiquitously and abundantly within infected cells. Their role in tumor biology and clinical outcomes across the spectrum of cancer is not fully explained. Here, we applied our bioinformatics pipeline for quantitative EBV miRNA detection to examine sequencing data of 8,955 individual tumor samples across 27 tumor types representing the breadth of cancer. We uncover an association of intermediate levels of viral miRNA with decreased survival in adult acute myeloid leukemia (AML) patients (P = 0.00013). Prognostic modeling of this association suggests that increased EBV miRNA levels represent an independent risk factor for poor patient outcomes. Furthermore, we explore differences in expression between elevated and absent viral miRNA loads in adult AML tumors finding that EBV positivity was associated with proinflammatory signals. Together, given no associations were found for pediatric AML, our analyses suggests EBV positivity has the potential for being a prognostic biomarker and might represent a surrogate measure related to immune impairment in adult patients.

## Introduction

Epstein Barr virus (EBV) is linked to 1.5% of all human cancers across the world^1^ based on known viral-containing malignancies such as Burkitt Lymphoma (BL), nasopharyngeal carcinoma (NPC), stomach cancer, and diffuse large B cell lymphoma (DLBCL). EBV appears integral to oncogenesis and virus containing tumors have been associated with distinct patterns of tumor mutations supporting the role of EBV as an oncogenic driver^2,3^. The full extent of EBV’s influence has been difficult to determine using genomics, and classification remains largely based on Epstein Barr virus encoded small RNAs (EBERs) staining of tumor samples^4^. Systematic genomic study of EBV and its effects on cancer prognosis through genome and transcriptome sequencing has been limited due to lack of sensitive and consistent detection methods^5^.

Investigations are also complicated by the chronicity of EBV infection. Following primary infection in an immune competent individual, EBV is controlled, and becomes a lifelong chronic infection. It lies latent inside a small percentage (1–10 cells per million) of host B cells, as well as possibly a subset of epithelial cells, with minimal protein translation, thereby escaping immune surveillance by the human host (reviewed in^6^). EBV persistence has been associated with a variety of host immune evasion strategies, including inhibition of immune cell function, dampening of apoptotic pathways, and interfering with antigen processing pathways. Moreover, since the virus has established a life-long persistence in 97% of adults globally, low levels of detectable virus in cancer do not necessarily imply infected tumor cells but might simply represent detection of a normal chronic infection^7^.

However, the virus may impact the microenvironment around infected cells and thus have the potential to influence the tumor without directly infecting malignant cells^8^. Also, the ability to control the virus and EBV reactivation may serve indirectly as a surrogate of general host immune competence. An example of such interplay is the inflammatory effects of EBV on gastric cancers through pro-inflammatory molecule expression such as cytokines responsible for increase in growth in EBV infected cells (IL5,IL6)^9^. Hence, EBV infection and its control has the potential to give insight into both tumor biology and host immunity.

To date, genomic and molecular studies across the breadth of cancer have been limited in their inclusion of viral content. While EBV has been observed by EBERs detection in tumors not traditionally thought to be infected, such as head and neck, lung, ovarian, colon, and esophageal cancer, these cancers have not been routinely screened for EBV^10^. There have also been reports of EBV positive tumors which lack significant EBERs expression^4^. While more data rich, genomic studies have focused on viral messenger RNA (mRNA) transcriptome evaluation, such studies are limited by the low levels of viral mRNA expression and have focused on further understanding the role of EBV in cancer cell biology. In addition, these were mainly limited to studying classic cases of EBV-associated malignancies, such as BL and NPC^2,11^. Other genomic studies are also confounded, including exome sequencing since probes for EBV capture are not routinely included limiting the ability of secondary analysis across a wide breadth of cancer.

Unlike viral messenger RNAs (mRNA), EBV microRNAs (miRNAs) were shown to be ubiquitously expressed during EBV latency and often make up a notable number of cellular miRNAs. Viral miRNAs comprise upwards of a quarter of all miRNAs in studied eBL lines such as Jijoye^12^. These miRNAs are a major mechanism by which the virus manipulates the host cells during latency^13^. Studies have suggested that EBV miRNAs were able to dampen immune surveillance of the infected host cells by down-regulating important genes in viral detection, such as the JAK/STAT pathway^8^. Moreover, EBV miRNAs in a few cancers have been associated with angiogenesis and more aggressive tumors^14–17^. Specific EBV miRNAs have also been linked to poor prognosis in patient outcomes. For example miR-Bart20-5p expression was associated with decreased survival in gastric cancer tumors^15^. Furthermore, EBV miRNA transcripts were also reported to be an independent prognostic indicator for patients diagnosed with advanced NPC^15,18^. More intriguing a recent study of approximately 2300 individuals from a more limited set of 13 cancers from TCGA study showed the presence of any EBV miRNAs was correlated with poor survival in low stage malignancies^16^.

Here, we aimed to systematically ascertain and quantify the presence of EBV within cancer types by screening for viral miRNA. To do so, we developed a computational framework for EBV miRNA detection and quantification using publicly available datasets. We then categorized viral miRNA expression levels as a means to identify EBV-infected tumors or loss of immune control over EBV in the tumor microenvironment and apply this metric in patient survival analyses finding an association with poor prognosis in adult AML, which we subsequently investigate further.

## Results

### EBV detection and classification across cancers

**Figure 1:**
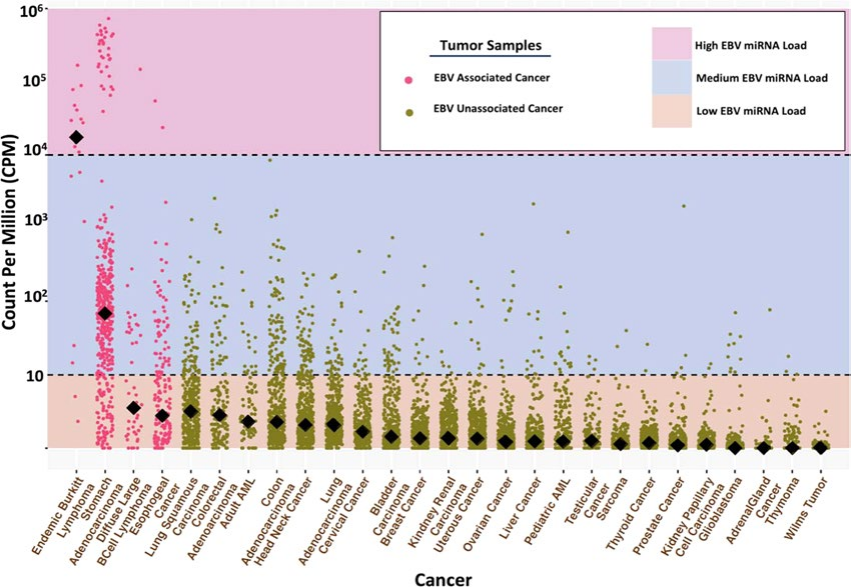
EBV miRNA quantitative expression across cancer spectrum. In general, the total viral miRNA load at the highest levels (≥10^4^ CPM) were observed only in tumors previously associated with harboring the virus (pink/purple), which correlate with reported proportions of viral positive tumors in the literature. Previously unassociated tumors (green) posses viral miRNA levels only at intermediate (10^1^–10^4^ CPM) or low (<10^1^ CPM) levels. Gastric tumors were interesting in that a majority (69.5%) fall within in the medium category suggesting an association with EBV beyond simple viral infection of the tumor in the 10% at high levels. The y-axis represents log10 counts of viral miRNA per total human and viral miRNA aligned to their respective genomes. The dashed lines separate the defined categories of viral miRNAs based on designated expression levels and and diamond lines denote median expression levels.

To better assess the presence of EBV across a spectrum of cancers, we examined 27 different tumor types for the presence of 44 EBV miRNAs as well as smaller fragments of other viral non-coding RNAs (ncRNAs) including EBERs (Methods and Supplementary Fig. S1). We included miRNA-seq datasets encompassing a total of 8,955 individual tumors with an average of 336 samples per cancer (range 19–592). The samples were predominantly drawn from the TCGA and TARGET datasets, and we also included 19 EBV-associated eBL tumors that our group has previously sequenced (Methods, Table 1)^14^. Tumor specimens, which might include other host cells within the tumor microenvironment, had marked differences in EBV miRNA abundance (Fig. 1). EBV presence was not solely correlated with past associations of viral positive tumor cells. Overall, samples with EBV miRNAs and EBERs ≥ 1 copies per million (CPMs) were present in 27% (n = 2418) of all samples. EBV positive samples ≥ 10 CPMs total viral miRNAs and EBERs were detected in 10.1% (n = 903) of all tumor samples with individual cancers ranging from 1% to 89% viral miRNA load. However, this did not represent necessarily infected tumor cells, given that the vast majority of adults who were latently infected with EBV were thereby harboring virus at low levels in latently infected B cells. Thus, detecting low levels of EBV was not unexpected, particularly given that up to 25% of an infected cells’ miRNA could be of viral rather than human origin^14^. We therefore aimed to set rational thresholds that would be indicative of tumor cell infection as well as levels that might define uncontrolled EBV-infected non-tumor cells within the tumor microenvironment.

**Table 1:** EBV miRNA levels for each cancer type.

Cancer	Total	Negative	Positive	Medium EBV Levels	High EBV Levels
Acute Myeloid Leukemia, Adult	103	88 (85%)	15 (15%)	15 (15%)	0
Acute Myeloid Leukemia, Pediatric	263	247 (94%)	16 (6%)	16 (6%)	0
Adrenal Gland Cancer	95	94 (99%)	1 (1%)	1 (1%)	0
Bladder Carcinoma	456	412 (9%)	44 (1%)	44 (10%)	0
Brain Cancer	501	491 (98%)	10 (2%)	10 (2%)	0
Breast Cancer	479	469 (98%)	10 (2%)	10 (2%)	0
Cervical Cancer	307	292 (95%)	15 (5%)	15 (5%)	0
Colon Adenocarcinoma	462	368 (8%)	94 (2%)	94 (2%)	0
Diffuse Large B Cell Lymphoma	45	30 (67%)	15 (33%)	14 (31%)	1 (2%)
Endemic Burkitt Lymphoma	19	2 (11%)	17 (89%)	5 (26%)	12 (63%)
Esophageal Cancer	186	135 (73%)	51 (27%)	49 (26%)	2 (1%)
Head and Neck Cancer	528	457 (87%)	71 (13%)	71 (13%)	0
Kidney Papillary Cell Carcinoma	291	289 (99%)	2 (1%)	2 (1%)	0
Kidney Renal Clear Cell Carcinoma	537	532 (99%)	5 (1%)	5 (1%)	0
Liver Carcinoma	377	366 (97%)	11 (3%)	12 (3%)	0
Lung Adenocarcinoma	522	487 (93%)	35 (6%)	35 (6%)	0
Lung Squamous Carcinoma	504	442 (88%)	62 (12%)	63 (12%)	0
Ovarian Cancer	592	570 (96%)	22 (4%)	22 (4%)	0
Prostate Cancer	499	496 (99%)	3 (1%)	3 (1%)	0
Rectal Adenocarcinoma	161	120 (75%)	41 (33%)	40 (24%)	0
Sarcoma	124	121 (98%)	3 (1%)	3 (2%)	0
Stomach Adenocarcinoma	456	105 (23%)	351 (76%)	317 (68.5%)	34 (7.5%)
Testicular Cancer	134	130 (97%)	4 (2%)	4 (3%)	0
Thymoma	124	122 (98%)	2 (2%)	2 (1.6%)	0
Thyroid Cancer	503	501 (99.5%)	2 (0%)	2 (0.5%)	0
Uterus Cancer	548	536 (98%)	12 (2%)	12 (2%)	0
Wilms Tumor	139	139 (100%)	0	0	0

Positive samples are samples with $\ge$10 counts per million (CPM) of viral miRNA and EBER expression. Negative samples are those with ≤10 CPM of viral miRNA and EBERs levels. Green lines signify cancers samples previously associated with Epstein Barr Virus (EBV). The first number on each cell of “number of samples with medium/high EBV level”, and the second number in the parenthesis represents “the percent of the latter samples”.

For this, we categorized three general groupings. We first defined a subset of cancers with abundant or high-level EBV miRNA expression at ≥10,000 CPM. These were likely a result of EBV containing tumors and miRNA expression within the tumor cells and represented 0.52% of the total samples examined. This category captured all cancers known to harbor EBV and at frequencies consistent with previous reports based on EBER positivity: 63% for eBL, 7.5% for stomach cancer, and 2.2% for diffuse large B cell lymphoma. These samples appeared by-and-large to express the full complement of viral miRNAs. While concordant with EBV positive rates as defined by EBERs staining, these levels were likely conservative. For eBL, positivity was lower than determined based on other metrics^2^. As no samples were categorized as high-level outside of tumors with previous EBV-associations, our results were consistent with other tumor types not directly harboring the virus, except for the possibility of rare sporadic cases or a small proportion of tumor cells.

The second level of expression we categorized as medium (10–10,000 CPM), which could be the consequence of a low fraction of infected tumor cells, lower expression within tumor cells, or benign B cells harboring the virus (circulating or tumor-infiltrating lymphocytes) reflective of poor immune control over the infection^13^. Overall, 10% (n = 898) of our samples fell within this category. In general, this correlated with tumors known to have significant lymphoid infiltrates such as gastrointestinal cancers. Interestingly, 68% (n = 321) of gastric cancers fall within the medium category and these appear to be distinct from the samples with high EBV miRNA abundances. Finally, 88% (n = 7863) of samples screened had low to absent EBV miRNA expression levels (<10 CPM). Such low levels likely represent EBV-negative tumors and individuals with well-controlled infections. This threshold of 10 CPM was consistent with our knowledge of tumors lacking an association with EBV and those minimally associated tumor infiltrating lymphocytes. This included tumors such as Wilms tumor, adrenal gland carcinoma where none of those we examined surpassed the 10 CPM cutoff. This level conservatively suggested that less than 1 in 100,000 cells were infected with EBV presuming viral miRNAs were 25% of total cellular content^19,20^.

### Pattern of miRNAs and EBERs across cancer subtypes

**Figure 2:**
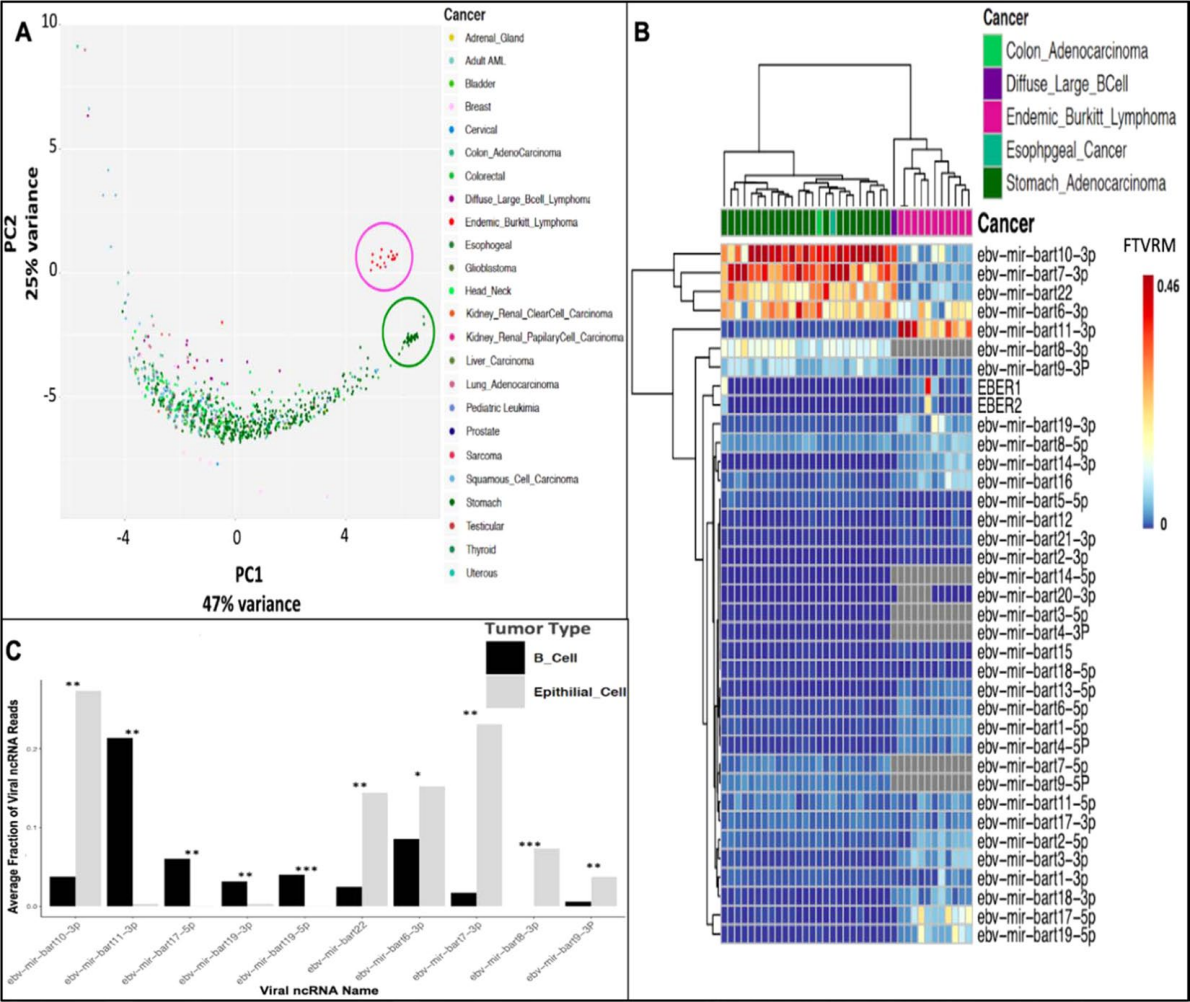
PCA on relative expression of EBV miRNA of EBV positive samples. (**A**) PCA1–2 represented 72% of the variance across samples. The pink circle included tumor samples with B cell origins (PC2). The green circle encompassed tumor samples with epithelial origins (PC1). (**B**) Heatmap of hierarchical clustering of the samples circled in (**A**) in terms of patterns of viral miRNA expression in total fraction of viral reads per million (FTVRM). (**C**) Bar plots represented cell type specific patterns of viral miRNAs expression across samples (from red circle and purple circled samples in **A**). Each bar represented average fraction of EBV miRNA expression. Note only P-value significant EBV miRNAs (P < 0.05 wilcoxon rank test with bonferroni correction) were shown with above average fraction of 0.03 per miRNAs across samples, bar plot of all viral miRNAs can be found in Supplementary Fig. 2C.

We sought to investigate the pattern of small RNA expression across cancers and performed principal component analysis (PCA) (Fig. 2A). We examined only samples with significant EBV content (≥10 CPM). The first three principal components (PCs) accounted for 90% of the variation (Fig. 2A and Supplementary Fig. S2). Most samples fell on a continuum defined by PC1 which appeared to correlate with the absolute amount of expression. PC1 and PC2 defined two distinct clusters of B cell derived (pink/purple) and epithelial (green) apart from the majority of sample. The eBL and diffuse large B cell lymphoma fell into what we defined as the B cell cluster while high level gastric and esophageal cancers fell in the epithelial cell cluster. We also compared the expression from these two clusters more closely revealing distinct expression patterns (Fig. 2B). ebv-mir-bart8-5p, ebv-mir-bart17-3p, and ebv-mir-bart11-5p were expressed in both B cells and epithelial cells. For highly expressed miRNAs (>1000 CPM) (Fig. 2C), we found ebv-mir-bart8-3p, ebv-mir-bart7-3p, ebv-mir-bart22, ebv-mir-bart10-3p, ebv-mir-bart6-3p and ebv-mir-bart9-3p are increased in epithelial cluster. Highly-expressed EBV miRNAs increased in the B cell cluster were ebv-mir-bart11-3p, ebv-mir-bart-17-5p and ebv-mir-bart19-3p, and ebv-mir-bart19-5p.ic. Given these significant differences are based on cell type, these may provide useful markers for better understanding the cell type of origin within tumors or provide specific biomarkers.

### Survival outcome for EBV positive tumor samples across cancer subtypes

**Figure 3:**
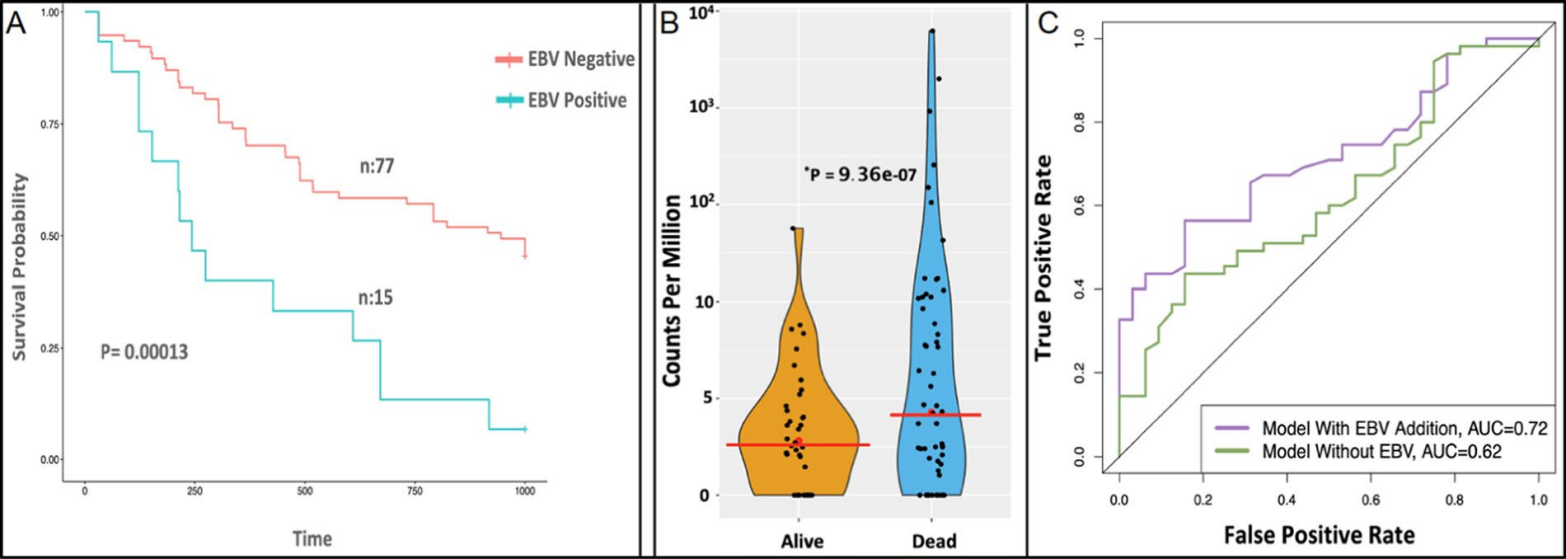
Survival analysis and prognostic model for EBV status in adult AML. (**A**) Kaplan-Meier curve compared the survival of EBV positive (blue) versus negative (red) AML patients in 1000 day survival analysis which significantly differed (P < 0.00013). (**B**) The difference in total miRNA EBV levels was remarkable when comparing patients who survived or died with only one survivor greater than 10^1^ CPM (wilcoxon rank test P-value = 9.3 × 10^−07^). (**C**) Area under the curve (AUC) based on cutoffs for the multivariate Cox prediction model using bootstrapped cross validation when EBV was modeled as a feature along with age, percent eosinophils, and number Trisomy 21 inversion 16 mutations.

To address our central question regarding whether EBV impacts the clinical outcome of cancer patients, we used our more sensitive measure of total EBV miRNA in our survival analysis. We examined patient survival across tumor types hypothesizing that levels above a normally controlled infection could be associated with clinical outcomes. Using both the Kaplan-Meier test and general linear model (GLM), we tested EBV positivity, defined by combining high and medium levels, as ≥10 CPM in comparison to negative tumors, defined as to low or absent expression (<10 CPM). Based on this categorization, only one tumor type, adults with acute myeloid leukemia (AML) demonstrated significantly different 1000 day survival rates when controlling for multiple testing. EBV positive adult AML patients had significantly worse survival rates relative to their EBV negative counterparts at 1000 days (P = 0.00013 Kaplan Meier, Bonferroni-corrected P = 0.02, Fig. 3A, Supplementary Table S1). For EBV positive patients survival for an adult AML diagnosis was only 6% (1 out of 15 EBV positive tumors) compared to 43% (31 out of 72) for patients with EBV negative tumors, with an odds ratio of 0.095 (0.012–0.76). This survival difference was robust even with a lower threshold (Supplementary Table S1). Moreover, patients who died had significantly higher EBV miRNA levels than those who survived (Fig. 3B). Interestingly, this association was not observed for pediatric AML where there was no difference between EBV status and survival (56% for EBV positive (n = 9/16) and 59% for EBV negative (n = 148/247), Kaplan Meier P = 0.74). The survival disadvantage in adults was robust even after controlling for sex and age (GLM P = 0.0439). EBV infection status alone was not significantly associated with other clinical and molecular risk factors, including AML French, American and British classification (FAB), based on univariate analysis using the 84 of 103 AML samples with detailed clinical annotation (Supplementary Table S2). Together, our findings suggest that EBV miRNA levels might represent an independent biomarker associated with poor prognosis in adult AML.

### Prognostic risk model for adult AML incorporating EBV miRNA

To examine the potential prognostic power of EBV miRNA status for survival in conjunction with other known risk factors, we performed multivariate Cox regression analysis (Table 2). Our multivariate analysis detected other previously associated factors including age, eosinophil levels, inversion 16 and trisomy 21 significantly associated with risk in adult AML tumors. EBV was the strongest prognostic predictor among all features. We also examined interactions between risk factors, such as age and percent eosinophils, to detect potential confounding. Our results indicated <1% confounding suggesting that EBV miRNA status was independently associated with survival in adult AML (Table 2). We next tested the prognostic power using a Cox model incorporating the above variables and previous risk factors along with EBV miRNA status. A machine learning algorithm was used to determine the significance of the addition of EBV miRNA positivity in the prognosis of AML patients. We again examined interactions and removed any highly correlated factors^21^ (Supplementary Fig. S3). Using bootstrapping and leave-one-out cross validation (LOOCV), we then tested the prognostic power on withheld samples from our Cox-model of survival incorporating EBV status as well as other significant univariate predictors (percent eosinophils within tumors, Trisomy 21, Inversion 16, and Age). The discriminatory power represented by the area under the curve (AUC) improved with the inclusion of EBV miRNA status as a variable (0.62 to 0.72 respectively) (Fig. 3C, Supplementary Table S3). To better assess the significance we permuted EBV status which showed that our observation of the AUC was significant (P = 0.02).

**Table 2:** Multivariable Cox regression for AML samples.

Feature	Coefficient	Pr(>|z|)
Age	2.22E−02	0.08207
EBV	1.32E + 00	0.0045
Eosinophil Percent	2.52E − 01	0.00457
Lymphocyte Percent	3.86E − 05	0.99648
Myelocyte Percent	3.96E − 02	0.55609
Neutrophil Percent	−6.65E − 03	0.62223
Monocyte Percent	2.09E − 03	0.95954
Translocation 8–21	−3.28E − 01	0.64948
Inversion 16	−2.34E + 00	0.03145
Translocation 8	9.62E − 02	0.87208
del(5q)/5d-	1.01E + 00	0.12995
del(7q)/7d-	7.36E − 01	0.25181
Translocation 9–22	−1.38E + 01	0.98869
Trisomy 21	2.75E + 00	0.00411
Translocation 4–11	−2.02E + 00	0.14934
Translocation 9–11	1.89E + 00	0.15012
Translocation 15–17	−1.39E + 00	0.16105
idh1_r140	1.20E − 02	0.98419
idh1_r132	−2.22E + 00	0.0387
NPMC	4.37E − 01	0.37311
FLT3	−5.90E − 02	0.88937
Gender	−4.17E − 01	0.30107

The coefficient represents the impact of features. Pr(>|z|) defines the statistical significance. |z| is the absolute value of the Wald statistics. It represents if the feature coefficient is statistically different from zero. Each translocation is from a chromosome to another, for example Translocation 8–21 is a translocation between chromosomes 8 and 21. The cytogenetic deletion on various sites and sizes on chromosome 7, and 5 are represented by del. Mutations on the *IDH1* gene on arginine amino acid (r) on various sites (132,140) observed in adult AML tumors. FLT3 mutation are consistently observed in forms of insertion, deletions and missense and intronic variants and reported as part of patient clinical information.

As an alternative model, akin to previous post remission treatment (PRT)^22^, we used the cytogenetic classification instead of the individual key cytogenetic aberrations. Although we were not able to replicate the previous PRT score with available data, due to lacking a measure of blast numbers within the TCGA dataset, we were able to demonstrate that EBV miRNA status incorporation improved the model: AUC 0.59 to 0.70 for poor and AUC 0.59 to 0.69 for intermediate outcome category (Supplementary Fig. S4, Supplementary Table S3). Overall, our modeling suggested that EBV miRNA levels have the potential to serve as a clinically actionable prognostic biomarker for adult AML.

### Host transcriptome expression difference between EBV positive and negative adult AML tumor samples

**Figure 4:**
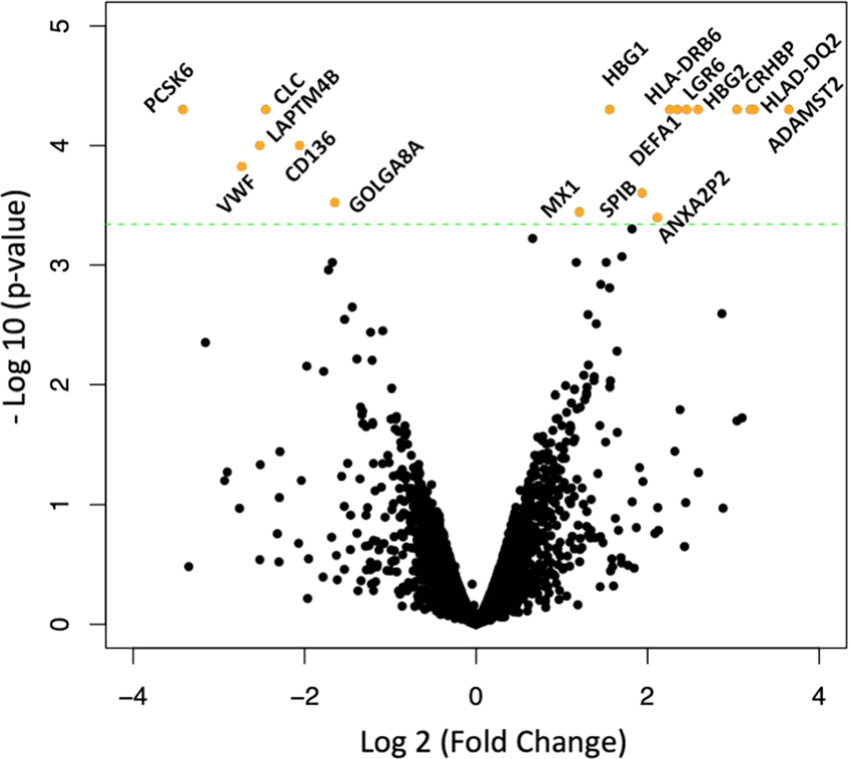
Differential mRNA transcriptome gene expression analysis comparing AML cases with medium and no EBV miRNA expression. Volcano plot showed differential expression between samples above 10 CPMs of EBV reads (n = 12) and those lacking viral miRNA and EBER expression (and n = 14). Genes increasing in EBV positive AML show a fold change increase (right side of the volcano plot) and those decreasing show a reduction in fold change (to the left side of the volcano plot). Genes which were colored orange posses q-values (false discovery rate) below 0.1 (Supplementary Table S4).

We next sought to explore potential biological differences that might underlie increased levels of EBV miRNA at diagnosis and help explain the association with decreased survival. We first performed differential transcriptome expression analysis on host mRNA expression in relation to EBV miRNA status (12 adult AML samples at EBV miRNA and EBERs 1000 > CPM ≥ 10 (medium EBV group), and 14 samples at CPM = 0 (no EBV reads), respectively) with available RNA-seq data. We detected 18 differentially expressed human genes (q ≤ 10%) after we removed genes that were also significantly differentially expressed when survivors and nonsurvivors were compared in these samples (Fig. 4). Host genes previously known to be upregulated during EBV infection of B cells were also upregulated in our dataset such as HLA-DQ and HLA-DRB^23^. A collection of genes associated with inflammation, interferon I and II activity in addition to neutrophil and macrophage deregulators were also differentially expressed between EBV positive and negative samples. These genes include MX1, ANAX2P2, DEFA1, NCF1C, CLC, and SPIB^24–27^. GO biological enrichment analysis found enrichments including an increase in reactive oxygen species and metabolic cell processing pathways when the tumor samples were classified as EBV positive (ROS metabolic process). The latter process has been well studied in terms of its association with inflammation and increase in cancer cells^28^. The host genes involved in this process were HBG1, HBG2 and NCF1C. GO cellular component enrichment shows higher than average expression of MHC Class II proteins complex involvement in addition to increase in cytoplasmic vesicle formation. Reactome pathway analysis showed enrichment in immune system signaling such as interferon signaling, alpha defensins and cytokine signaling (FDR corrected P value ≤ 0.1) (Supplementary Table S5).

No viral mRNAs were observed to be relatively enriched, which was not unexpected given the normally low expression level of EBV poly A transcripts and the reduced or silenced viral gene expression during latency. In fact, we could barely detect viral mRNA reads (<7 reads per kilobase per million (RPKM) per gene) in 13 out of 15 EBV miRNA positive AML samples (Supplementary Fig. S5) with the greatest combined viral mRNA expression of 14 RPKM. The viral genes most evident were A73/RPMS1 and EBNA1, consistent with a latency I pattern of infection while EBV lytic genes including BALF4 and BNLF2A were minimially expressed. Two AML samples’ mRNA expression was constrained to two latency III genes, i.e. EBNA-2 and EBNA-LP although we could detect a mosaic of lytic genes expressed in the rest of the AML samples. The latter finding was consistent with our miRNA data which implied viral detection by the host immune system and upregulation of the host defense mechanisms during episodic lytic reactivation.

## Discussion

Ninety seven percent of the world’s adult population is latently infected with EBV yet it is not fully understood why certain individuals develop EBV-associated cancers. Here we used ubiquitously-expressed EBV miRNAs as a more sensitive metric, not only to detect EBV but also to quantify the virus residing within the tumors and/or the tumor microenvironment. Characterizing a wide variety of cancers for EBV miRNA levels, we found a novel association with elevated viral miRNA levels and poor survival in adult AML patients that likely represents an independent prognostic predictor. As EBV is a prevalent chronic infection we set reasonable cutoffs to differentiate tumors bearing the virus (high levels), in contrast to uncontrolled infection of normal and/or a small proportion of tumor cells (medium levels), and well-controlled or uninfected individuals (low or absent levels). In general, our results were consistent in identifying EBV within tumors with previously defined associations. Interestingly, miRNA expression found at medium levels in a wider variety of cancers suggest that EBV might be poorly controlled within the patient or within the tumor microenvironment. In support of this premise, the latter also correlated with tumor types known to contain higher proportions of tumor infiltrating lymphocytes^7,10,29^.

Given our novel association between EBV miRNA levels and adult AML survival, we focused on further exploring its potential as a prognostic biomarker as well as exploring its role as a surrogate for immune dysregulation. Using a multivariate Cox model EBV miRNA status appeared to be an independent biomarker for survival, further improving outcome predictions. We examined this both with de novo modeling as well as modeling based on the previously validated PRT models. While this association and its ramifications require additional validation in an independent cohort, it would be interesting to determine whether other measures of EBV, such as peripheral viral miRNA loads or peripheral miRNAs, might serve as surrogates of tumor burden, immune competence or able to provide longitudinal information during the course of treatment and while in remission.

To explore the biology of this association, we examined expression differences between patients with medium level viral miRNA expression compared to those with no evidence of EBV. We found that increased expression of innate inflammation mediators correlated with miRNA EBV levels. While this finding could be affected by confounding given AML represents an aberrant leukocyte precursor, one of the most interesting genes showing expression differences was MX1. MX1 gene has been linked, not only to EBV, but to progression of acute lymphocytic leukemia by acting as a binding partner of LMP1 viral gene in EBV positive samples in acute lymphocytic leukemia but as tumor inducing factor in myeloid leukemia^30^. MX1 has been shown to be involved in interferon alpha and interferon gamma expression and has been associated with reactivation of EBV infection in gastric cancer and lymphomas^31^.

Taken together, we proposed a model whereby EBV miRNA levels in adult AML patients reflect the immune status of the patient. Immune alterations during cancer could be general (and proceed tumorigenesis) or might be specific to the local tumor microenvironment. This would be consistent with the lack of association in pediatric AML, where increased EBV may due to primary infection or reactivation directly rather than immune dysfunction. Further exploration examining EBV from peripheral blood as well as isolated tumors and tumor infiltrating lymphocytes (using single cell RNA-seq) could help answer such questions. Our lack of association in pediatric AML might be in part due in part to the relative lack of children being infected with EBV compared to adults, but we hypothesize that EBV in children may represent primary infections or reactivations that are unassociated with decline in immunity.

We also found that expression patterns of viral miRNAs are differentiated based on tumor cell type, broadly separating them into B cell derived tumors and those of epithelial origin (Fig. 2C). Of the highly expressed EBV miRNAs we found differential expression where ebv-mir-bart11-3p, ebv-mir-bart-17-5p, ebv-mir-bart19-3p, and ebv-mir-bart19-5p were increased in B cell tumors; whereas ebv-mir-bart8-3p, ebv-mir-bart-7-3p, ebv-mir-bart22, and ebv-mir-bart10-3p were increased in epithelial tumors. These findings are consistent with previous studies^32–34^. For instance, ebv-mir-bart19-5p has been reported to downregulate LMP1 expression^35^ which is not expressed in EBV latency I malignancies (that do not express LMP1) compared to epithelial tumors that have a latency II viral gene expression pattern (which by definition expresses LMP1). Also, ebv-mir-bart22 has been observed to suppress LMP2 expression and protect the infected cells from immune recognition^35^. This viral miRNA was higher in EBV latency II epithelial gastric tumors, which was consistent with the normal cell type latency pattern^36^. It will be interesting to further investigate EBV miRNA signatures in terms of tumor cell type of origin and determine if viral self-regulation mechanisms also promote tumorigenesis (ie cell survival). It will also be interesting to see if B cell versus epithelial signatures can be further correlated with EBV sub-localization within the tumor microenvironment. While speculative, given we did not find pronounced signatures for B cell tumors with medium expression, further investigation into the roles of shared EBV miRNAs should provide insight into the mechanisms of EBV tumorigenesis, regardless of malignant cell type.

Overall, our findings of prevalent miRNAs, elevated above levels observed in controlled infection, provide evidence that EBV may represent an important prognostic biomarker and can provide important insight into host immune status associated with survival. Further validation, as well as extending these findings to peripheral markers and determining whether monitoring EBV miRNA levels during the course of disease may further predict adverse long-term outcomes, are warranted. Most importantly our results indicate that the investigation of EBV both biologically and as a biomarker should not simply be limited to instances where cancer cells contain the virus.

## Materials and Methods

### Tumor sample selection and analysis

Tumor miRNA sequencing (miRNA-seq) and associated clinical data was obtained from The Cancer Genome Atlas (TCGA) (https://cghub.ucsc.edu) and Therapeutically Applicable Research to Generate Effective Treatments (TARGET)(https://ocg.cancer.gov/programs/target) projects through dbGaP (Project #13089) and combined with miRNA and RNA sequencing of eBL from Western Kenya (dbGaP accession number phs001282.v2.p1)^14^. For each tumor type up to 592 miRNA tumor samples were chosen, preferentially selecting those with available messenger RNA sequencing (mRNA-seq)^37^. Overall, a total of 8,955 miRNA samples were analyzed. All adult AML were collected as primary tumors and no information about future chemotherapy regimen was provided in the clinical metadata for these samples. Nasopharyngeal cancer (NPC) samples were not added to this study due to the absence of EBV reads in the available public miRNA seq datasets.

### Ethical approval and sample collection

Fine-needle aspirates (FNA) were obtained between 2009 and 2012 at the time of diagnosis and prior to commencing chemotherapy at Jaramogi Oginga Odinga Teaching and Referral Hospital (JOOTRH), the regional referral hospital for pediatric cancer located in western Kenya. Giemsa/May-Grünwald was performed on the FNAs for morphologic diagnosis by microscopy. All experiments were performed in accordance with relevant guidelines and regulations. Written informed consent was obtained from all subjects or, if subjects are under 18, from a parent and/or legal guardian before enrollment. Ethical approval was obtained from the Institutional Review Board at the University of Massachusetts Medical School (UMMS) and the Scientific and Ethics Review Unit at the Kenya Medical Research Institute.

### Detection and quantification of EBV miRNAs

The miRNA binary alignment map (bam) reads were extracted and adapter sequences removed using Cutadapt-1.9.1. The trimmed reads were then aligned simultaneously to the human (hg19) and EBV reference genome (NC_007605) to detect best placements using Burrows-Wheeler Aligner (bwa-0.7.17). This alignment was done with recommended miRNA-aligning parameters including zero mismatch for the seed of 8 base pairs. This protocol was optimised for miRNA-seq but additionally detected other viral ncRNAs including EBERs^38–40^. To remove human contamination including cross mapping human miRNAs, only miRNA alignments that best mapped to the EBV genome were retained. We allowed for multiple best placements within the EBV genome so as not to exclude miRNAs arising from the repetitive regions. The method allowed for one base differences relative to the reference genome. Specific miRNAs and small EBER fragments were then quantified based on their defined genomic locations (miRBase version 22) as copies per million (CPM) using bedtools and in-house parsing scripts^41–43^. We did not detect significant expression of novel miRNAs outside miRBase defined locations. Total counts per million (CPM) values of all samples are now added as (Supplementary Table S6) for de-identified samples.

### Survival analysis

Survival analyses were performed in R including Kaplan-Meier survival analysis (using survival package), as well as univariate and multivariate logistic regression. Exclusion of potential confounders was determined by measuring the association before and after adjusting for a potential confounding variable and subsequently excluding those variables with greater than 10% change of effect^44^.

### Hazard analysis using cox regression with bootstrapping and cross validation

In order to assess the risk associated with the hazard of EBV positivity in our dataset, we used the standard Cox multivariable risk assessment method to regress the parameters of interest jointly. The R scripts for Cox-regression, bootstrapping and risk prediction using cross validation could be accessed through GitHub (https://github.com/bailey-lab/EBV_Cox-Regression.git) utilizing the censboot and Cox-regression packages using the general multivariate estimator g(x,ß) = e^xß^ in R. To test prognostic power, we used a multivariate 1000 bootstrapped leave-one-out cross validated (LOOCV) model to predict the risk associated with being EBV positive in concordance with patient survival, whereby we estimated the risk of EBV positivity of our parameters^45,46^. Area under the curve (AUC) for the performance of our prediction model was estimated at various thresholds to ascertain the risk associated with EBV for every predicted sample and permutation was used to determine the significance of AUC curves.

### Differential expression analysis

Differential expression analysis comparing EBV positive versus EBV negative samples was performed using the Tuxedo Tools (cufflinks, and cuffmerge and cuffdiff). Gene ontology (GO) enrichment analysis was done using python in house scripts utilizing Panther database^47^.

Data was visualized using a variety of R packages including libraries ggplot2, qqman, prcomp, dplyr, Tydiverse and pheatmap. All statistical analyses were done using validated R, and python functions and scripts.

## Supplementary information

All Supplemental Figures

All Supplemental Tables

## Data Availability

No data was generated during the current study. Tumor samples analysed for this study were downloaded from The Cancer Genome Atlas (TCGA) and Therapeutically Applicable Research to Generate Effective Treatments (TARGET)projects through dbGaP (Project #13089) and combined with miRNA and RNA sequencing of eBL from Western Kenya (dbGaP accession number phs001282.v2.p1).
