# Supplementary material for: Sensitive detection of EBV microRNAs across cancer spectrum reveals association with decreased survival in adult acute myelocytic leukemia
Source: Sci Rep. 2019 Dec 30;9:20321. doi: 10.1038/s41598-019-56472-1 (PMC6937232; doi:10.1038/s41598-019-56472-1)
Supplement: Supplementary file 1 — All Supplemental Figures [file 41598_2019_56472_MOESM1_ESM.docx]

**Supplementary Figure Legends**

**Sensitive detection of EBV microRNAs across cancer spectrum reveals association with decreased survival in adult acute myelocytic leukemia**

Mercedeh Movassagh^1^, Cliff Oduor^2,3^, Catherine Forconi^4^, Ann M. Moormann^4^, Jeffrey A. Bailey^1,5^

^1^ Department of Bioinformatics and Integrative Biology, University of Massachusetts Medical School, Worcester, MA, USA

^2^ Center for Global Health Research, Kenya Medical Research Institute, Kisumu, Kenya
^3^ Department of Biomedical Sciences and Technology, Maseno University, Maseno, Kenya
^4^ Department of Medicine, University of Massachusetts Medical School, Worcester, MA, USA
^5^ Department of Pathology and Laboratory Medicine, Warren Alpert Medical School, Brown University, Providence, RI, USA


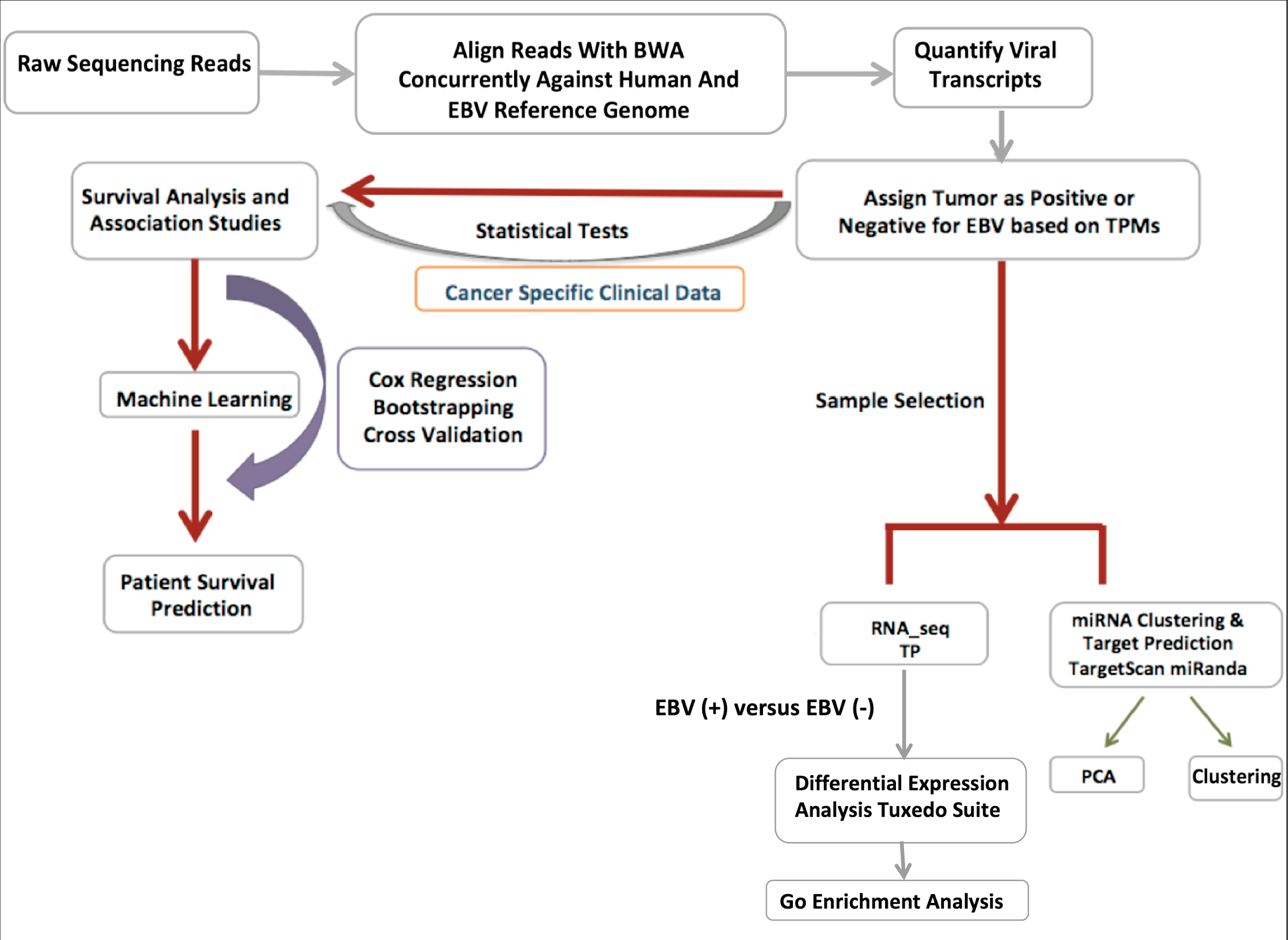


[**Supplementary Figure S1**](https://docs.google.com/document/d/1md-ri1OVwRnDrjzBy-aG_wHhqka4rt6I_izctqIEdZE/edit?disco=AAAACm7r5S4&ts=5c8999dd&usp_dm=false#sufig_Pipeline)

**Overall pipeline for EBV identification and our downstream analysis.** miRNA seq data was downloaded from TCGA, TARGET or our own cohort of eBL samples. The files were reverted to fastq and aligned to a concatenated human and EBV genome to insure the best alignment match and limiting multi-mapping reads. The viral non-coding RNA loci was assigned to the mapped reads and counts per million of the viral reads to the total library was estimated. Hereafter, statistical clinical associated followed by machine learning was utilized to understand survival association. Additionally, the detected EBV positive samples in comparison to those with no viral reads detected were further examined for mRNA level differences and miRNA variations across samples.


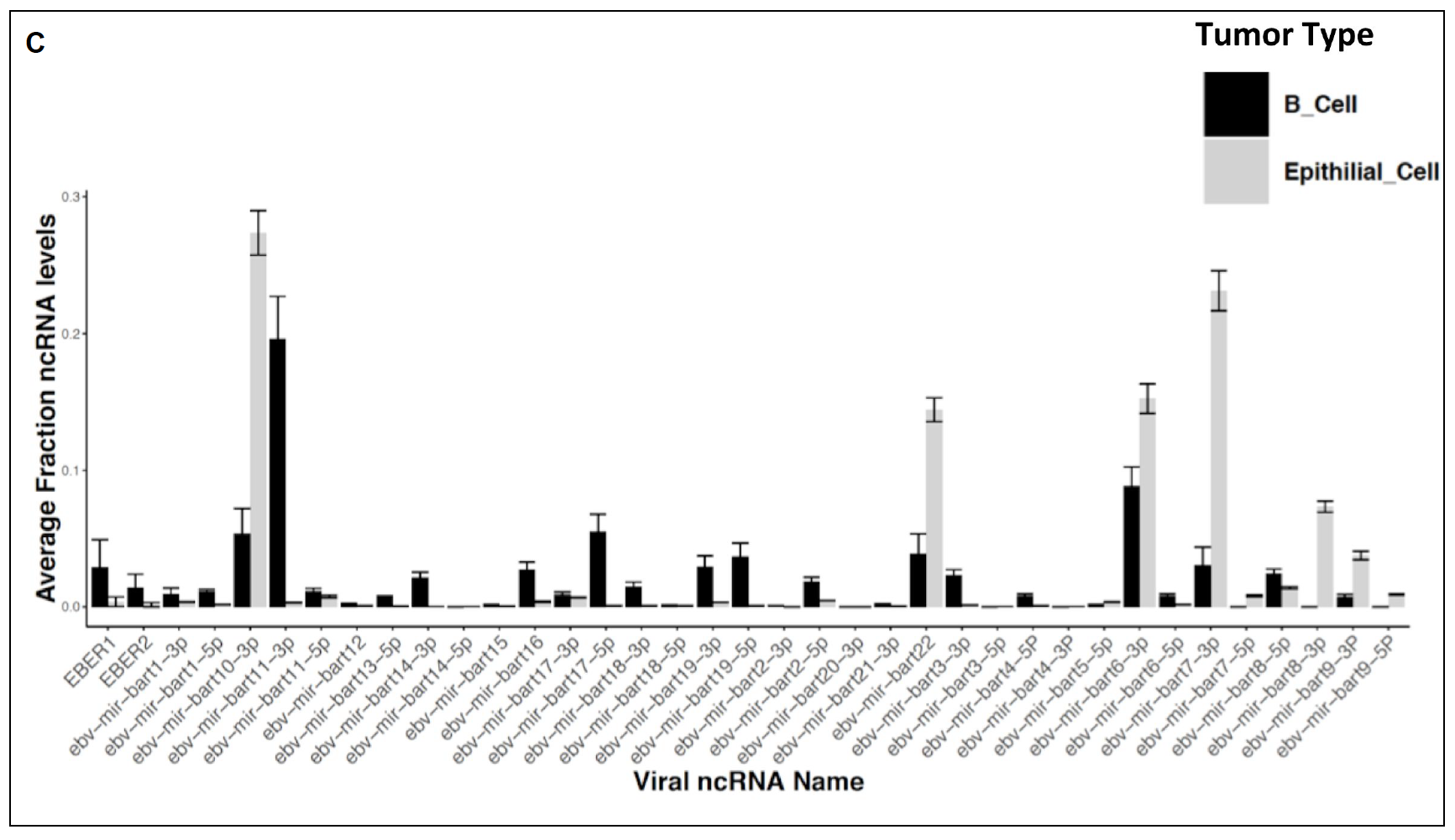

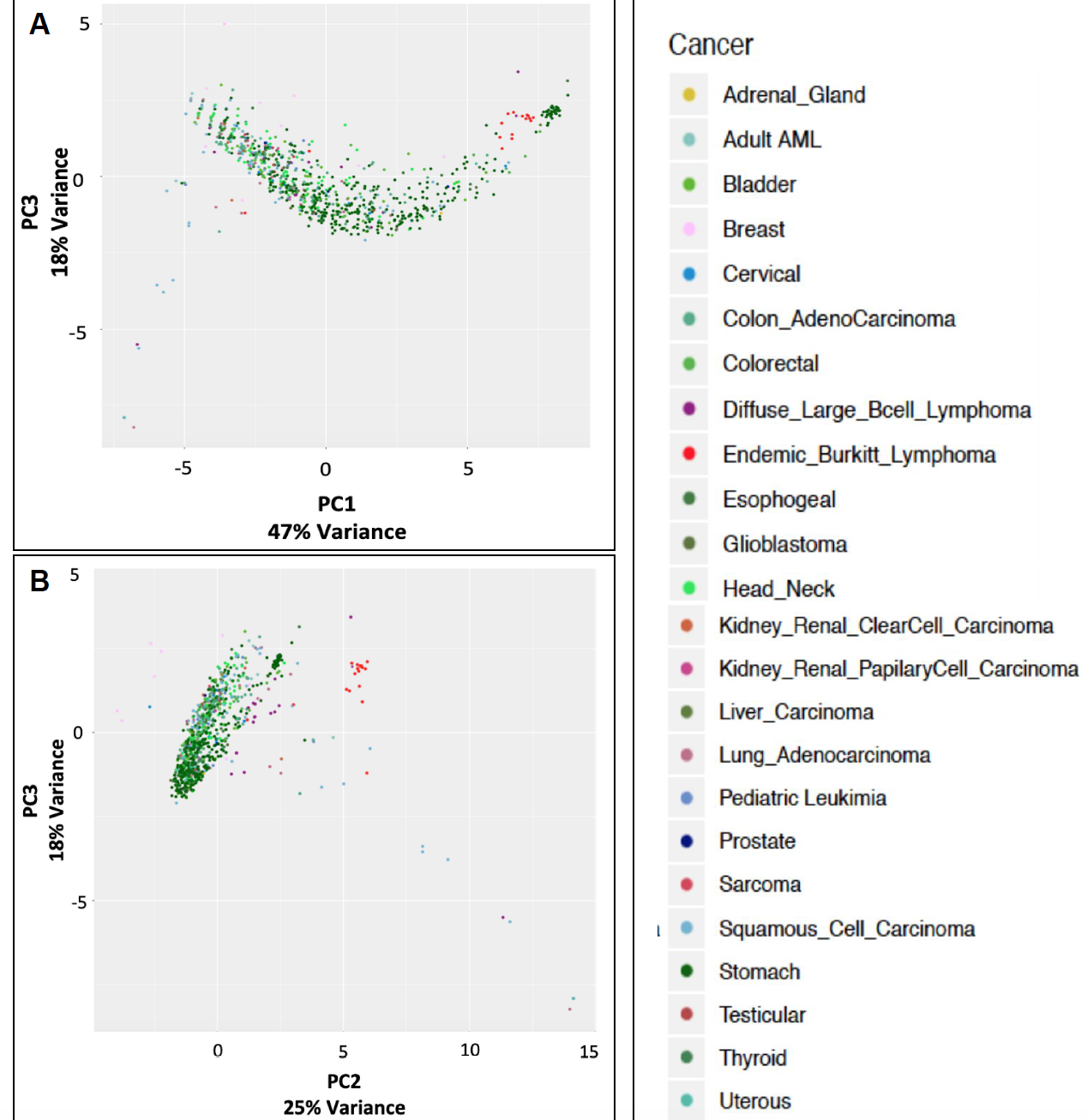


[**Supplementary Figure S2**](https://docs.google.com/document/d/1md-ri1OVwRnDrjzBy-aG_wHhqka4rt6I_izctqIEdZE/edit?disco=AAAACm7r5S4&ts=5c8999dd&usp_dm=false#sufig_PCA_Bar)

**Patterns of EBV miRNA expression across cancer subtypes. A.** PC1 versus PC3 **B.** PC2 to PC3 (each dot is a sample; the color represents the cancer subtype) **C.** Bar plot showing expression of all viral miRNAs across the cell type specific groups most similarly clustered based on PCA.

**
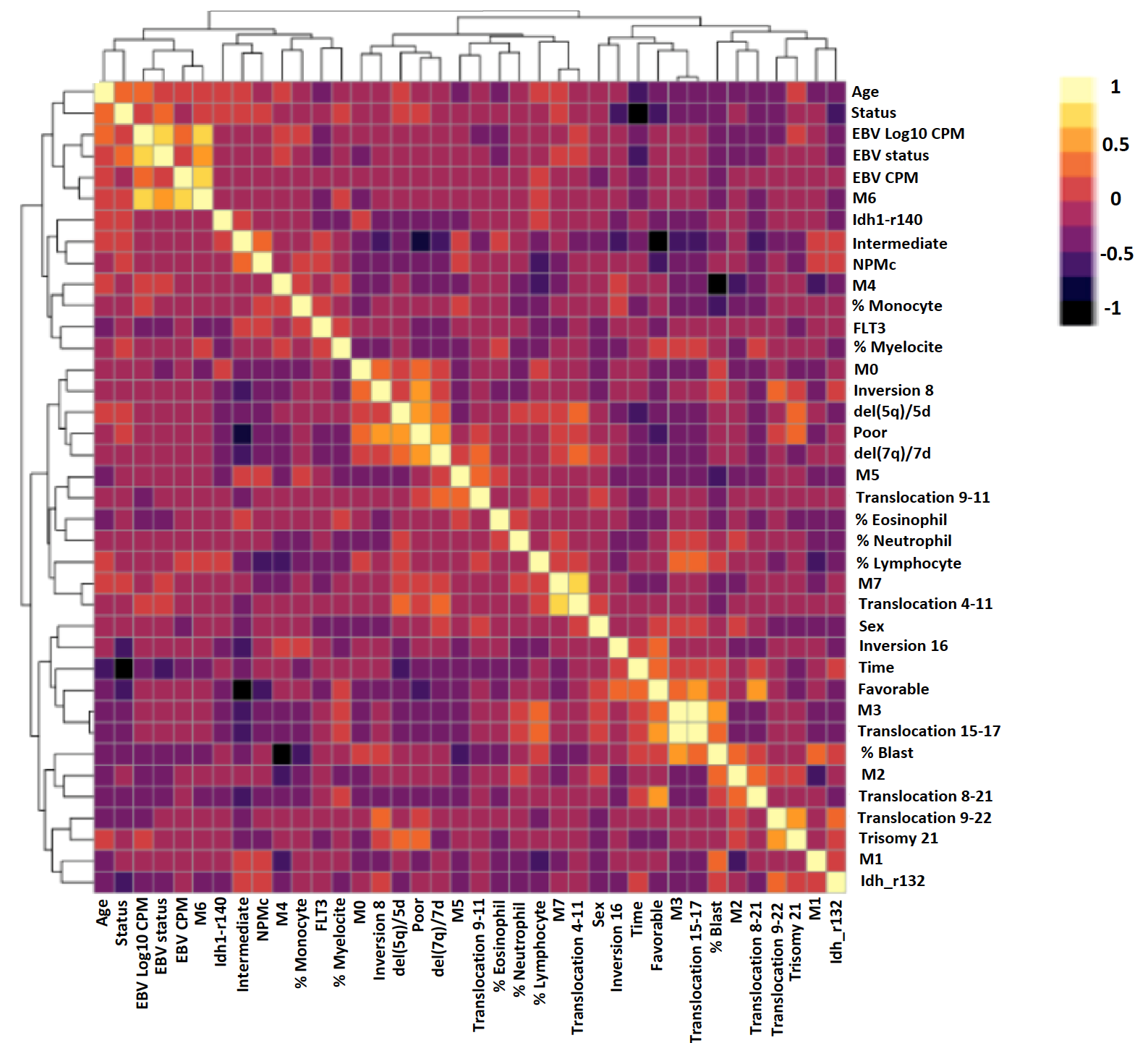
**

[**Supplementary Figure S3**](https://docs.google.com/document/d/1md-ri1OVwRnDrjzBy-aG_wHhqka4rt6I_izctqIEdZE/edit?disco=AAAACm7r5S4&ts=5c8999dd&usp_dm=false#sufig_MLAUCs)

**Cox regression model parameterization.** 2 Spearman correlation of various clinical factors including EBV smRNA reads.


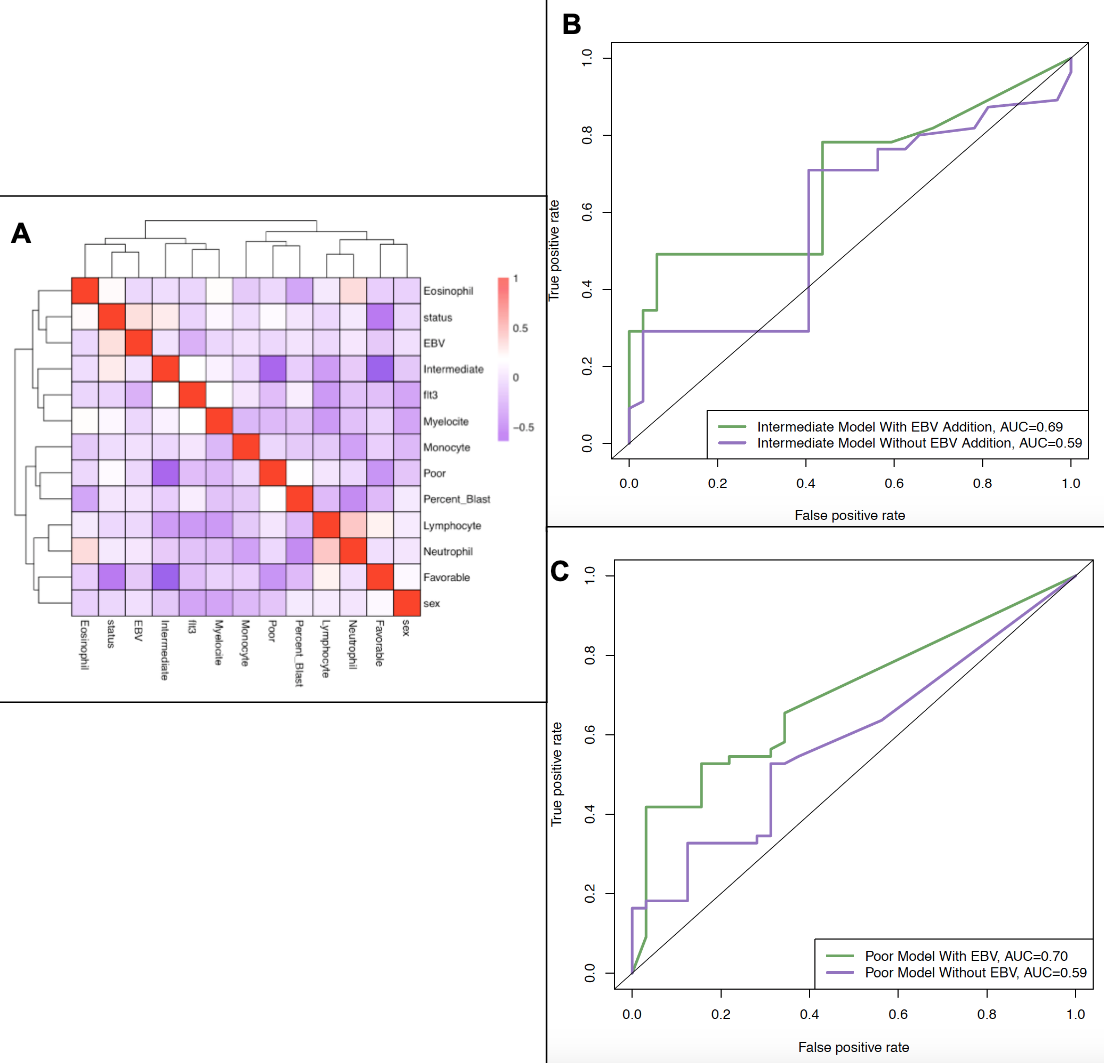


[**Supplementary Figure S4**](https://docs.google.com/document/d/1md-ri1OVwRnDrjzBy-aG_wHhqka4rt6I_izctqIEdZE/edit?disco=AAAACm7r5S4&ts=5c8999dd&usp_dm=false#sufig_modelCyto)

**Cox regression on cytogenetic classification of Adult AML. A.** Multivariate cox – regression model with cytogenetic outcome factors as the co-variants. **B & C.** Area under the curve (AUC) curves for the model if cytogenetic factors were used as the main strata with and without EBV present in the model.


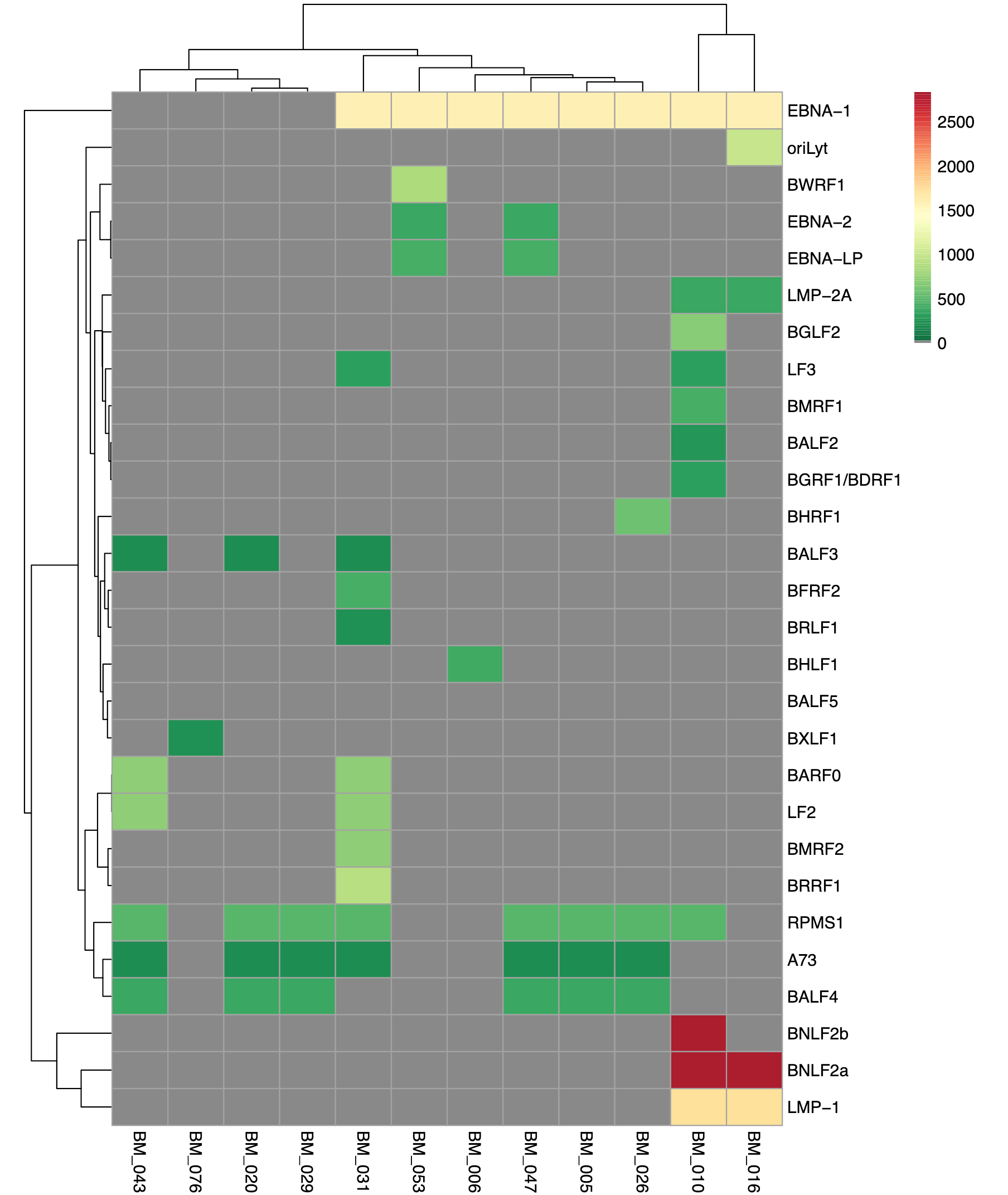


[**Supplementary Figure S5**](https://docs.google.com/document/d/1md-ri1OVwRnDrjzBy-aG_wHhqka4rt6I_izctqIEdZE/edit?disco=AAAACm7r5S4&ts=5c8999dd&usp_dm=false#sufig_EBVmRNA)

**Viral mRNA expression in adult AML patients.** EBV mRNA reads per kilobase of transcripts per million (RPKM) across EBV positive adult AML patients. Viral mRNA expression of 12 EBV positive samples based on RNA seq analysis. Grey represent viral mRNAs not expressed in the samples.
